# Supplementary material for: Botulinum Toxin Treatment of Adult Muscle Stem Cells from Children with Cerebral Palsy and hiPSC-Derived Neuromuscular Junctions
Source: Cells. 2023 Aug 15;12(16):2072. doi: 10.3390/cells12162072 (PMC10453788; doi:10.3390/cells12162072)
Supplement: Supplementary file 1 [file cells-12-02072-s001.zip › cells-2482679-supplementary.pdf]

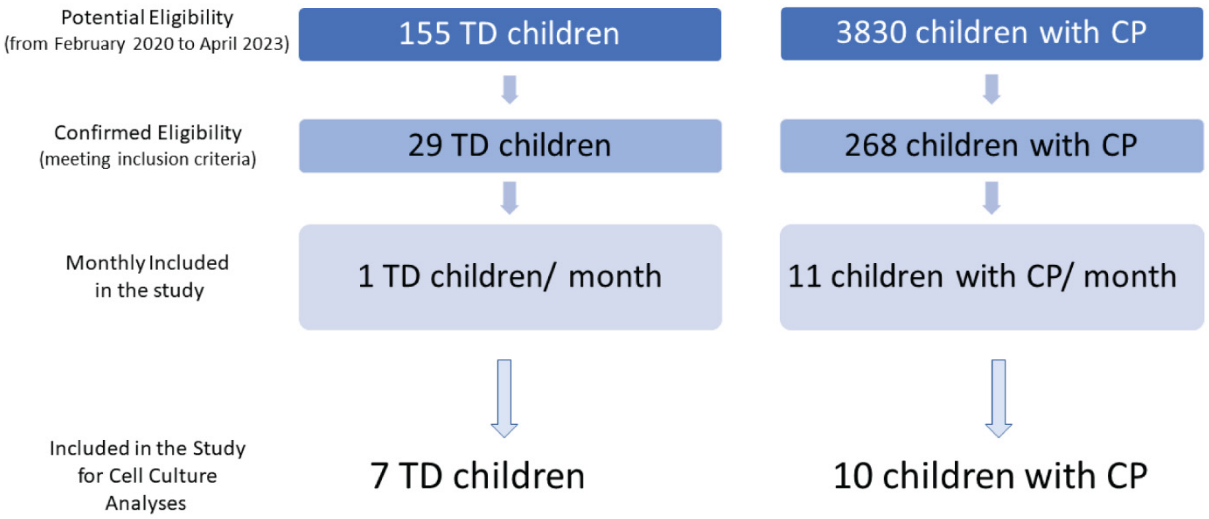

Figure S1: Flow chart representing the numbers of individuals during each stage study and the number of the sub-group recruited for cell culture experiments.

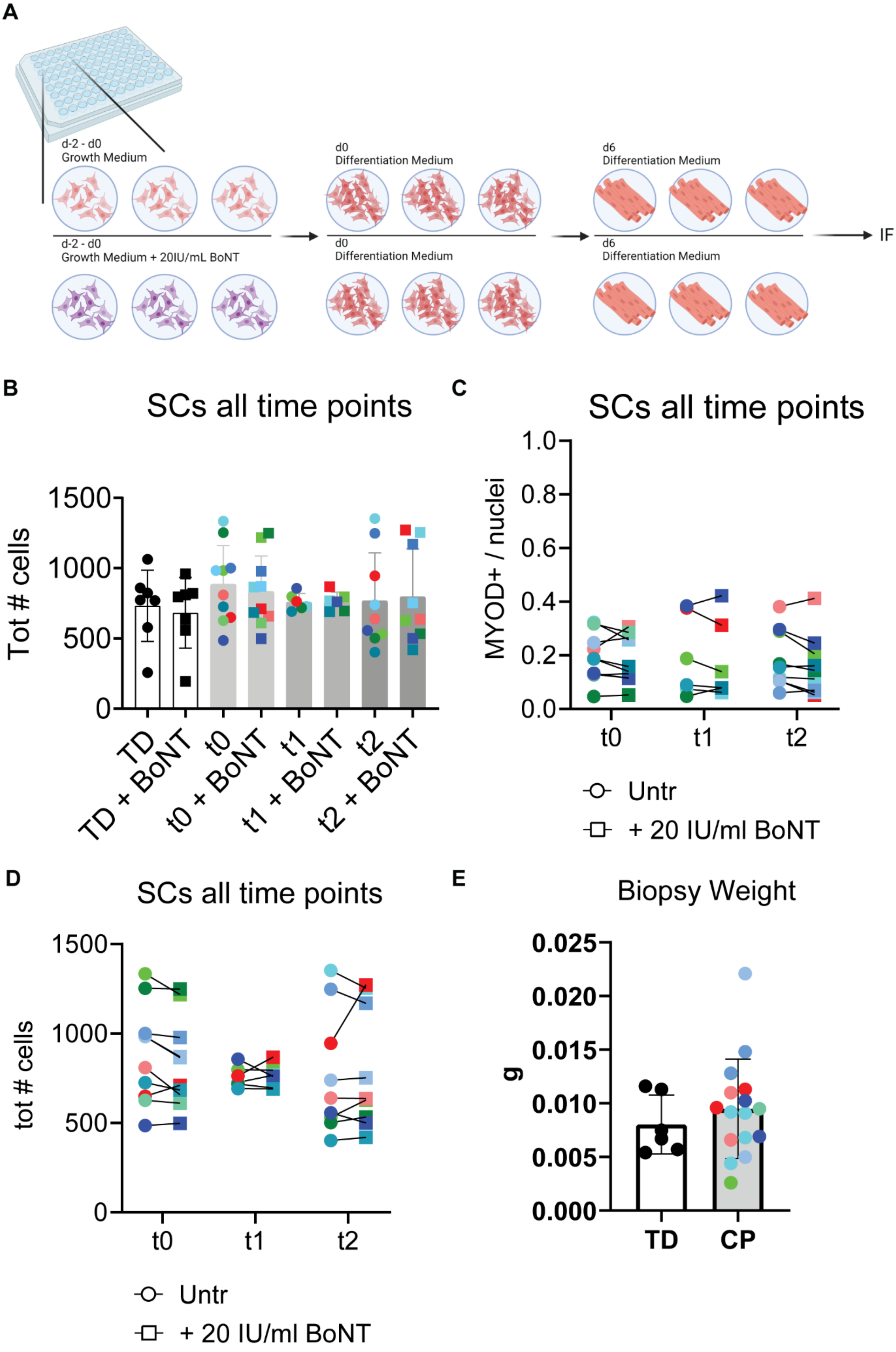
A
d-2 - d0
Growth Medium
d0
Differentiation Medium
d6
Differentiation Medium
d-2 - d0
Growth Medium + 20IU/mL BoNT
d0
Differentiation Medium
d6
Differentiation Medium
IF
B
SCs all time points
Tot # cells
1500
1000
500
0
TD
TD + BoNT
t0
t0 + BoNT
t1
t1 + BoNT
t2
t2 + BoNT
C
SCs all time points
MYOD+ / nuclei
1.0
0.8
0.6
0.4
0.2
0.0
t0
t1
t2
Untr
+ 20 IU/ml BoNT
D
SCs all time points
tot # cells
1500
1000
500
0
t0
t1
t2
Untr
+ 20 IU/ml BoNT
E
Biopsy Weight
g
0.025
0.020
0.015
0.010
0.005
0.000
TD
CP

Figure S2: Myogenic differentiation of SC-derived myoblasts treated with BoNT

(**A**) Schematic representation per patient of BoNT treatment (20 IU/ mL during 48h) on SC-derived myoblasts. In all conditions, cells obtained from each patient were seeded at the same density and the day after treated during 48h with 20 IU/ mL BoNT or maintained in normal growth medium. Two days later, differentiation medium was applied for 6 days, changing medium every two days. Representation of (**B**) total number of nuclei for TD (n = 7) and CP t0 (n = 10), time 1 (t1, 3 months after in vivo BoNT administration; CP n = 6) and time 2 (t2, 6 months after in vivo BoNT administration; CP n = 9) SC-derived myoblasts , (**C**) MYOD+ fractions and (**D**) total number of cells per field of view from children with CP at time 0 (t0, immediately before in vivo BoNT administration; CP n = 10), time 1 (t1, 3 months after in vivo BoNT administration; CP n = 6) and time 2 (t2, 6 months after in vivo BoNT administration; CP n = 9). Untreated samples were represented as round shaped symbols, while treated ones (20 IU/ mL BoNT for 48h) as squared shaped symbols. The same color highlights cells from the same sample, different tonalities of green are indicating GMFCS-I samples, of blue GMFCS-II samples and of red GMFCS-III samples. Data were analysed via Two-Way ANOVA followed by a Tukey post-hoc test: $*p < 0.05$ for t2 vs t0. (**E**) Biopsy weight (g) for the majority of the biopsies for TD and children with CP included in this study. The same color highlights biopsies from the same sample using different tonalities of green for GMFCS-I samples, of blue for GMFCS-II samples and of red for GMFCS-III samples. The same color highlights repetitive samples over time.

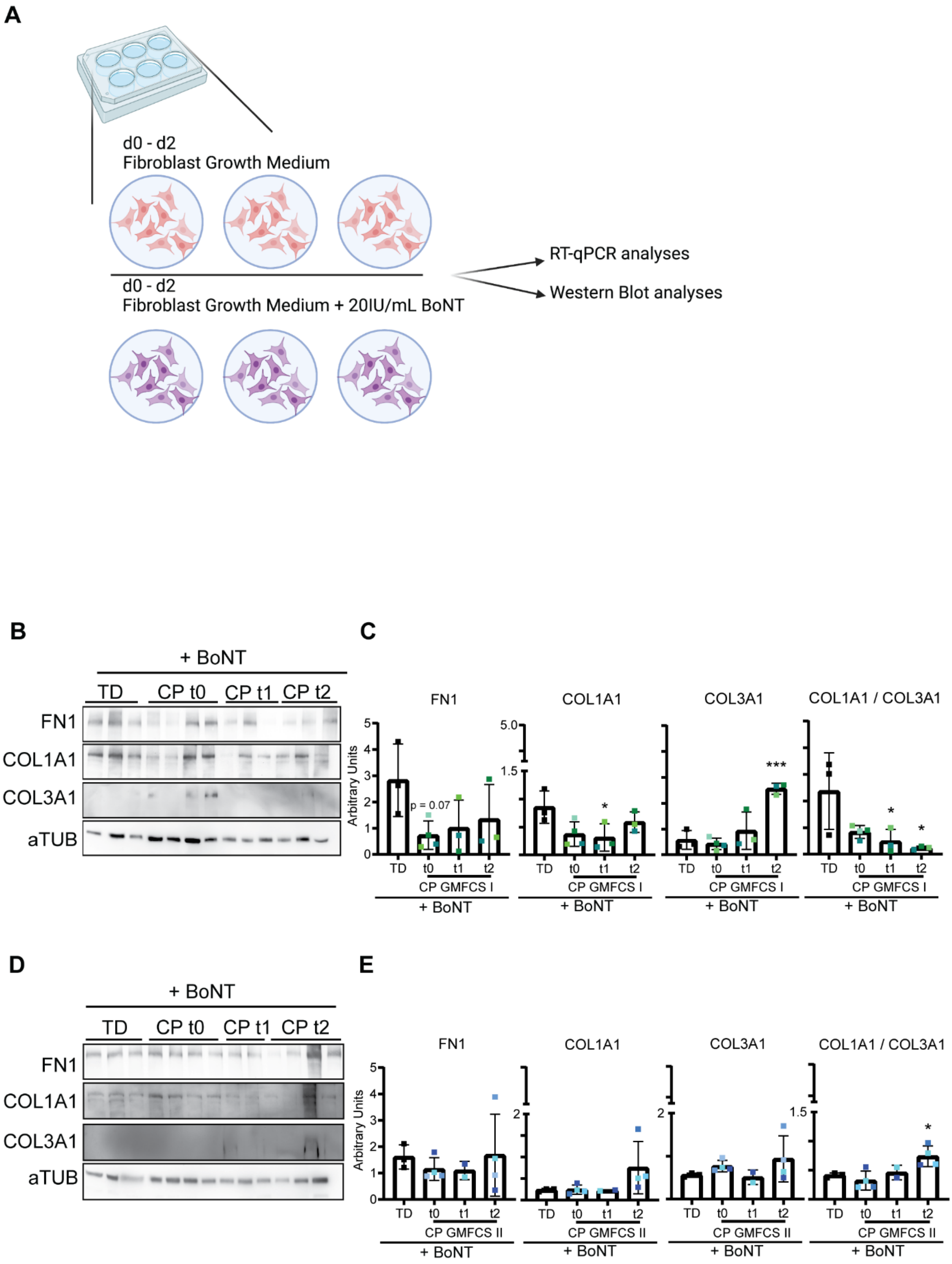

Figure S3: Treatment of muscle-derived fibroblasts with BoNT.

(**A**) Schematic representation of the treatment of muscle-derived fibroblasts with BoNT (20 IU/ mL during 48h). Cells were seeded at the same density and the day after treated with BoNT during 48h or maintained in normal growth medium. Two days later, cells were harvested for qRT-PCR or WB. (**B**) WB analysis and (**C**) protein level quantification for FN1, COL1A1 and COL3A1, normalized for aTUB expressed by 20 IU/ mL BoNT-treated fibroblasts extracted from TD and GMFCS-I CP samples at t0 (immediately before *in vivo* BoNT administration), t1 (3 months after *in vivo* BoNT administration) or t2 (6 months after *in vivo* BoNT administration). The same color highlights cells from the same sample, different tonalities of green for GMFCS-I samples and of blue for GMFCS-II samples. Data were analysed via One-Way ANOVA followed by a Tukey post-hoc test: $*p < 0.05$ for TD vs CP t1 or $***p < 0.001$ for TD vs CP t2. (**D**) WB and (**E**) protein level quantification for FN1, COL1A1 and COL3A1, normalized for aTUB expressed by 20 IU/ mL BoNT-treated fibroblasts extracted from TD and GMFCS-II CP samples at t0 (immediately before *in vivo* BoNT administration), at t1 (3 months after *in vivo* BoNT administration) or at t2 (6 months after *in vivo* BoNT administration). Treated samples (20 IU/ mL BoNT) were represented as squared shaped symbols. The same color highlights cells from the same sample, different tonalities of green are representing GMFCS-I samples and of blue for GMFCS-II samples. Data were analysed via One-Way ANOVA, followed by a Tukey post-hoc test: $*p < 0.05$ for CP vs TD, t1 or t2.

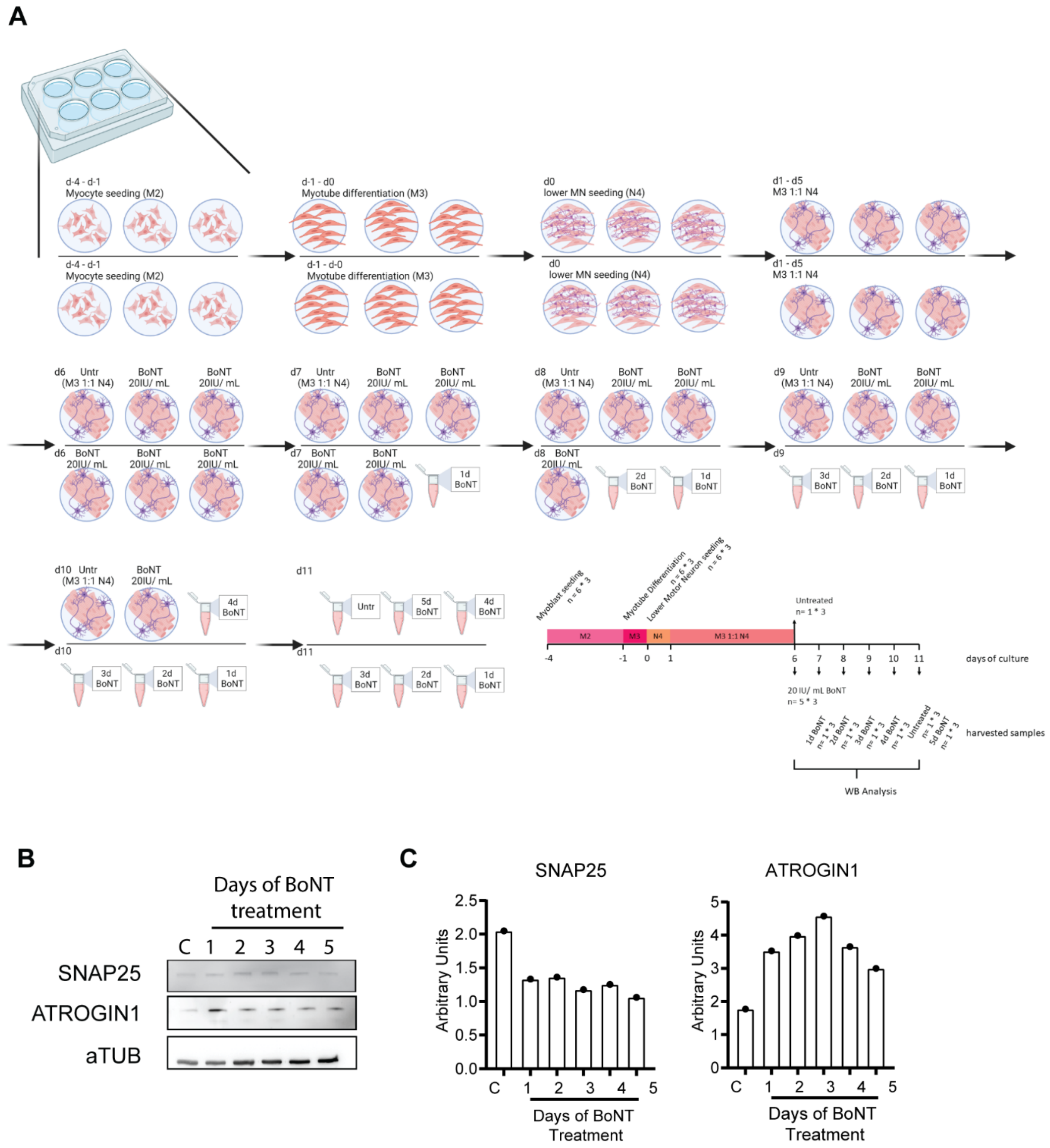

Figure S4: Visual and schematic representation of hiPSC-derived NMJs continuously treated with BoNT

(**A**) Visualisation and schematic representation of the plating and treatment of hiPSC-derived NMJs with BoNT (20 IU/ mL during 48h). Co-cultures were prepared by seeding 10.000 myocytes/ cm^2 kept in proliferating medium (M2; d-4 to d-1) for 2 days and inducing myocytes to differentiation the day after (d-1 to d0), with M3 medium. At day 0, 15.000/ cm^2 lower MNs

were seeded on top of myotubes in N4 medium, in order to favor their attachment and proliferation. From this point, a mix of M3 medium 1:1 with N4 medium was applied and medium was changed every other day. Treatment with 20IU/ mL BoNT (dissolved in a mix of M3 1:1 N4 medium) started at day 7 and last till day 11, when the 5-days BoNT treatment cells were detached together with the untreated cells. From day 7 to day 11, every day, one well of treated cells was pelleted for WB analysis. (**B**) WB and (**C**) quantification of SNAP25 and ATROGIN1 protein levels normalized by the levels of aTUB for untreated or 20 IU/ mL BoNT-treated cells during 1, 2, 3, 4 and 5 days. Data were representative of independent experiments.

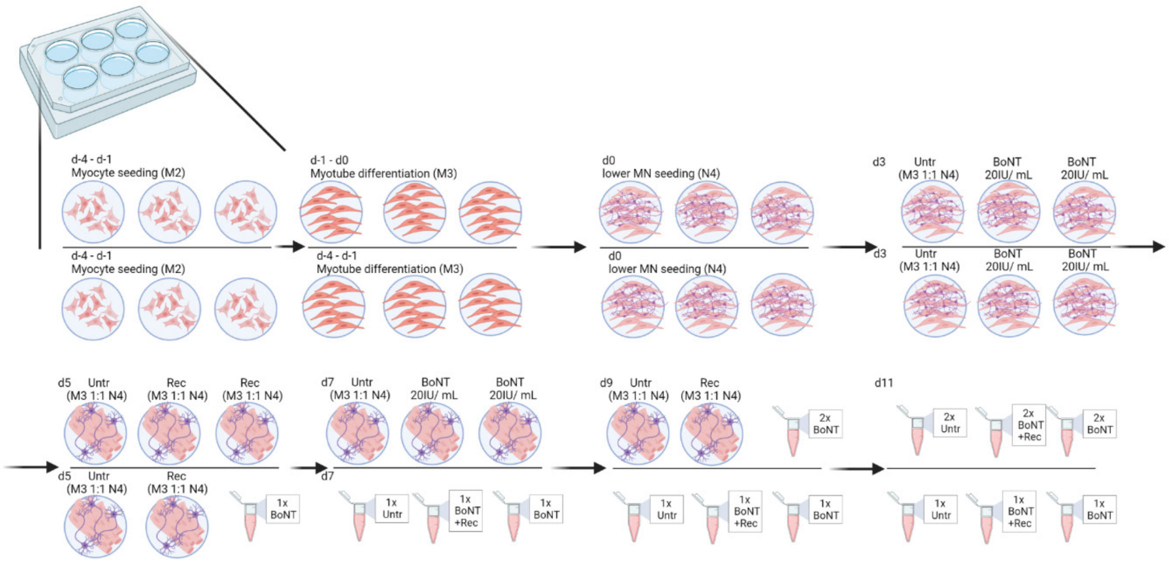

Figure S5: Visual and schematic representation of hiPSC-derived NMJs repeatedly treated with BoNT

Consecutive exposures of NMJ co-cultures to BoNT were tested to better mimic CP administrations. Co-cultures were prepared by seeding 10.000 myocytes/ cm^2 kept in proliferating medium (M2; d-4 to d-1) for 3 days and inducing myocytes to differentiation the day after (d-1 to d0), with M3 medium. At day 0, 15.000/ cm^2 lower MNs were seeded on top of myotubes in N4 medium, in order to favor their attachment and proliferation. From the day after, a mix of M3 medium 1:1 with N4 medium was applied and medium was changed every other day. The experiment started at day 3 on six wells, where 48h 20 IU/ mL BoNT treatment was applied on four wells, while untreated wells received a mix of M3 1:1 N4 medium (Untr). At day 5, one well was detached (indicated as 1x BoNT) and the other five wells were kept for further 2 days with normal M3 1:1 N4 medium. At this point, after detaching 1x untreated and 1x BoNT+Rec wells (day 7), the cycle was repeated with 48h 20 IU/ mL BoNT on two of the remaining wells. After this (day 9), detachment of one well (2x BoNT), and further 48h culturing with normal medium M3 1:1 N4 medium were applied. At the end of the experiment (day 11), the remaining two wells (indicated as '2x Untr' and '2x BoNT+Rec') were pelleted.

The experiment was repeated with the three cell lines available, always creating autologous co-cultures with autologous lower MNs and myotubes and treating six wells in the same standardized way for all cell lines.

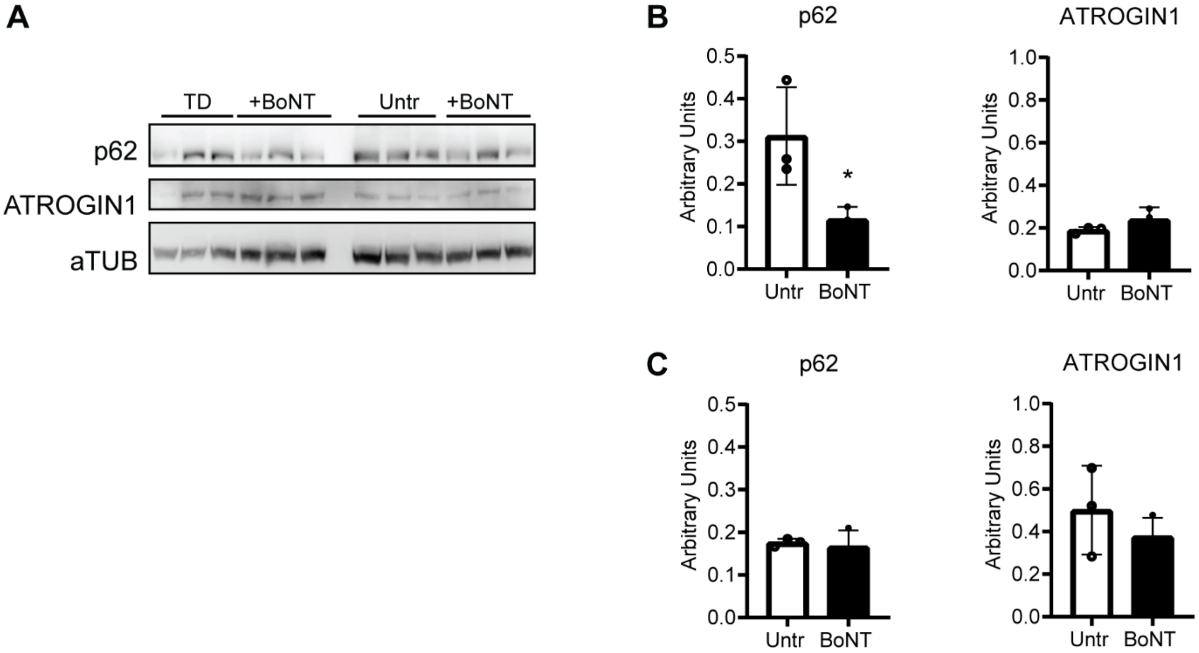

Figure S6: Protein degradation in TD SC-derived myotubes or hiPSC-derived myotubes treated with BoNT

(**A**) WB and protein level quantification for p62 and ATROGIN1, normalized for aTUB expressed by (**B**) untreated and BoNT-treated TD SC-derived myotubes and (**C**) untreated and BoNT-treated hiPSC-derived myotubes. Data were representative of independent experiments and values were expressed as mean ± SD. Data were analysed via t-test: *$p < 0.05$ for BoNT-treated cells vs untreated (Untr) ones.
